# Editorial: Digital implant dentistry: new developments to enhance clinical workflows and patient care

**DOI:** 10.3389/froh.2026.1913208

**Published:** 2026-07-21

**Authors:** Ethan Ng, John Rong Hao Tay, Jeniffer Perussolo

**Affiliations:** 1 Department of Restorative Dentistry, National Dental Centre Singapore, Singapore, Singapore; 2 Centre for Oral Clinical Research, Institute of Dentistry, Faculty of Medicine and Dentistry, Queen Mary University of London, London, United Kingdom; 3 Health Services and Systems Research Programme, Duke-NUS Medical School, Singapore, Singapore

**Keywords:** artificial intelligence, CAD-CAM (computer-aided design and computer-aided manufacturing), computer aided implantology, digital implant dentistry, digital treatment planning, GPT, machine learning

## Introduction

Over the past two decades, implant dentistry has become progressively digital. Computer-assisted treatment planning, guided implant surgery, intraoral scanners and computer-aided design and manufacturing (CAD/CAM) have transformed implant surgery and prosthesis fabrication, improving the efficiency of clinical workflows compared with conventional approaches (1, 2). More recently, large language models have expanded the application of artificial intelligence beyond image analysis, including clinical documentation and patient communication (3). This Research Topic explores how these technologies are transforming patient care and implant dentistry, while highlighting areas where further evidence is needed. The contributions span the implant care continuum, from data acquisition and surgical execution to information generation and communication.

Two papers examined the accuracy and feasibility of digital implant workflows in challenging clinical situations. Full-arch edentulous impressions are demanding scenarios in digital implant workflows, because the absence of anatomical landmarks and long inter-implant spans can increase stitching errors and cross-arch distortion. Gianfreda et al. found that auxiliary geometric devices attached to scan bodies and reported reduced centroid and total body root mean square deviations within clinically acceptable limits, without requiring additional scanning steps (Gianfreda et al.). However, improvements were limited to positional trueness, while angular accuracy did not improve significantly. This finding is consistent with a recent systematic review showing that such devices most reliably reduce linear (positional) deviation, while effects on angular accuracy remain less consistent and scanner-dependent (4). As the study was conducted *in vitro* using a single scanner system, further clinical validation is necessary. Zhang et al. described a fully digital workflow for a 73-year-old patient with essential tremor affecting the jaw and tongue, a condition that complicates both scanning of the edentulous arch and guided implant surgery (Zhang et al.). Six implants were placed using digital planning and a stacked surgical guide, resulting in marked improvements in patient-reported outcomes after one year. Although limited to a single case, the report illustrates how digital technologies may be particularly valuable in managing complex patients who might otherwise be unsuitable for fixed implant-supported rehabilitation. More broadly, it suggests that the greatest benefits of digital workflows may lie in overcoming clinical and functional challenges rather than simplifying routine cases.

Two further studies examined the use of artificial intelligence (AI) in generating dental information. Tay et al. evaluated four prompt-engineering strategies for answering common patient questions about dental implants (Tay et al.). All produced generally high-quality responses, judged across several domains including accuracy, clarity, source citation, and usefulness. Important trade-offs nonetheless emerged. The guideline-grounded (contextualised) model produced the most reliable citations but scored lower for clarity and relevance and performed least well on treatment-related questions. These findings suggest that authoritative sourcing alone does not ensure effective communication for lay audiences, an important consideration as more patients seek oral health information online before consultation. Yon, Ng and Bornstein reviewed the emerging literature on AI-generated dental reports (Yon et al.). Across studies involving panoramic radiographs and voice-transcribed clinical charting, natural language processing and fine-tuned models generated radiology reports and patient-facing explanations that were accurate for common findings and comparable in readability to human-authored reports, while improving patient-rated clarity. However, limitations included small validation datasets, limited language diversity, untested patient comprehension, and potential oversimplification of information. These observations reflect a broader challenge facing AI in healthcare, where the field must expand proof-of-concept performance toward robust evaluation in real-world clinical settings.

Collectively, the studies in this Research Topic illustrate the expanding role of digital technologies across implant care. Whereas earlier developments primarily focused on improving the accuracy and efficiency of treatment planning, guided implant placement, impression-taking, and prosthetic fabrication (5), newer innovations increasingly extend beyond procedural workflows to support risk assessment, outcome prediction, patient communication, and clinical decision-making (6). This shift reflects a transformation of the field, with digital technologies evolving from procedural tools into enablers of integrated and patient-centred care. Realising this potential will require prospective clinical validation of these digital and AI-based tools in real-world patient care, standardised outcome measures, and multilingual datasets representative of varied patient populations, health-literacy levels, and care settings to ensure that benefits are distributed equitably across healthcare systems rather than confined to well-resourced groups. Future research should also move beyond technical performance metrics to evaluate outcomes that matter directly to patients, including treatment experience, understanding, quality of life, access to care, and long-term implant success.

A common theme emerging from these studies is that the next phase of digital implant dentistry will be defined not simply by the digitisation of implant-related procedures, but by the ability of digital systems to support clinical decisions, workflows, and communication. Digital and AI tools tend to optimise what is easiest to measure, such as deviations in recorded implant positions (7), yet such technical metrics are often only proxies for outcomes that matter to patients. The positional accuracy of an implant impression is not the same as prosthetic success, and a fluent, well-sourced AI-generated patient answer or radiology report is not the same as clinical judgement or genuine patient understanding. Likewise, predictive algorithms and automated systems should be viewed as tools that augment, rather than replace, clinician expertise (8, 9). The true value of digital technologies may therefore lie less in replacing analogue steps and more in helping clinicians make better decisions, communicate more effectively, and deliver increasingly personalised care.

In conclusion, the future of implant dentistry will depend not on automation alone, but on human-centred technologies that combine computational capability with clinical experience, ethical oversight, and patient preferences. Ultimately, the value of digital implant dentistry should be judged not by the sophistication of its technologies, but by their ability to improve decision-making, patient experiences, and long-term treatment outcomes. Digital implant dentistry is not a novelty for its own sake, but a means of delivering care that is simultaneously more precise, more personalised, and more patient centred. The work presented in this Research Topic marks progress toward that goal while highlighting the evidence still needed to achieve it responsibly.
